# Binder-Free Zinc–Iron Oxide as a High-Performance Negative Electrode Material for Pseudocapacitors

**DOI:** 10.3390/nano12183154

**Published:** 2022-09-11

**Authors:** Qasim Abbas, Abdul Mateen, Abdul Jabbar Khan, Gaber E. Eldesoky, Asim Idrees, Awais Ahmad, Elsayed Tag Eldin, Himadri Tanaya Das, Muhammad Sajjad, Muhammad Sufyan Javed

**Affiliations:** 1 Department of Intelligent Manufacturing, Yibin University, Yibin 644000, China; 2 Beijing Key Laboratory of Energy Conversion and Storage Materials, Department of Physics, Beijing Normal University, Beijing 100084, China; 3 College of Chemical Engineering, Huanggang Normal University, Huanggang 438000, China; 4 Chemistry Department, College of Science, King Saud University, Riyadh 11451, Saudi Arabia; 5 Department of Applied Sciences, National Textile University, Faisalabad 37610, Pakistan; 6 Departamento de Quimica Organica, Universidad de Cordoba, E14014 Cordoba, Spain; 7 Faculty of Engineering and Technology, Future University in Egypt, New Cairo 11835, Egypt; 8 Centre of Excellence for Advance Materials and Applications, Utkal University, Bhubaneswar 751004, Odisha, India; 9 College of Chemistry and Life Sciences, Zhejiang Normal University, Jinhua 321004, China; 10 School of Physical Science and Technology, Lanzhou University, Lanzhou 730000, China

**Keywords:** binder-free, ZnFe_2_O_4_, nanoflakes, anode, supercapacitor

## Abstract

The interaction between cathode and anode materials is critical for developing a high-performance asymmetric supercapacitor (SC). Significant advances have been made for cathode materials, while the anode is comparatively less explored for SC applications. Herein, we proposed a high-performance binder-free anode material composed of two-dimensional ZnFe_2_O_4_ nanoflakes supported on carbon cloth (ZFO-NF@CC). The electrochemical performance of ZFO-NF@CC as an anode material for supercapacitor application was examined in a KOH solution via a three-electrode configuration. The ZFO-NF@CC electrode demonstrated a specific capacitance of 509 F g^−1^ at 1.5 A g^−1^ and was retained 94.2% after 10,000 GCD cycles. The ZFO-NF@CC electrode showed exceptional charge storage properties by attaining high pseudocapacitive-type storage. Furthermore, an asymmetric SC device was fabricated using ZFO-NF@CC as an anode and activated carbon on CC (AC@CC) as a cathode with a KOH-based aqueous electrolyte (ZFO-NF@CC||AC@CC). The ZFO-NF@CC||AC@CC yielded a high specific capacitance of 122.2 F g^−1^ at a current density of 2 A g^−1^, a high energy density of 55.044 Wh kg^−1^ at a power density of 1801.44 W kg^−1^, with a remarkable retention rate of 96.5% even after 4000 cycles was attained. Thus, our results showed that the enhanced electrochemical performance of ZFO-NF@CC used as an anode in high-performance SC applications can open new research directions for replacing carbon-based anode materials.

## 1. Introduction

High-speed industrial growth, the limited availability of fossil fuels, and fast-growing energy resource requirements need specially designed and immensely efficient energy storage devices for a viable future [1,2]. The development of energy storage has become a major and progressive research endeavor in both the commercial and community sectors as a result of the significant energy shortage [3]. Therefore, high-performance and novel energy storage and conversion devices are increasingly important. These devices, including batteries and supercapacitors (SCs), have attracted much attention from researchers to overcome the energy crisis [4]. SCs are emerging as cutting-edge green energy storage technology among all these energy storage devices in the modern age. In addition, compared with typical capacitors and batteries, SCs have a fast charging–discharging time, extended life cycle, higher power density, and suitable capacitance. These outstanding characteristics of SCs make them a favorable candidate for energy storage applications in electric cars, compact devices (such as cell phones, modern digital cameras, and laptops), energy management, and commercial power supplies [5].

According to the energy storage mechanism, SCs have two families, known as electrical double-layer capacitors (EDLCs) as well as pseudocapacitors (PCs). The former type is generally made of carbon-based materials and stores energy through the fast adsorption/desorption of electrolyte ions [5]. On the other hand, PCs are mainly assembled by transition metal oxides or sulfides and conducting polymers with a good electrochemical capacity [2,6]. PCs can store energy through reversible redox reactions in between the interfaces of electrode/electrolyte [7]. In the fabrication of novel electrode materials for PCs, the charge storage mechanism plays a crucial role to obtain an overall good level of performance. Compared with EDLCs, metal oxides revealed better electrochemical performance due to the redox reactions. Recently, bimetallic oxides, including, ZnMn_2_O_4_ [8], NiFe_2_O_4_ [9], ZnCo_2_O_4_ [10], NiCo_2_O_4_ [11], and ZnFe_2_O_4_ [12] have received much attention. ZnFe_2_O_4_ has been a promising electrode material for SCs because of its natural abundance, non-toxic nature, high specific capacitance, and cost-effectiveness [12,13,14]. However, as it is mainly studied as the positive electrode of SCs, it is essential to explore its performance as a negative electrode material.

The two types of electrode materials (anode and cathode) are used to fabricate high-performance SCs. Therefore, in-depth investigations have been carried out to synthesize positive electrode (cathode) materials using cost-effective and fast approaches. Compared with cathode materials, significantly less consideration has been given to negative electrode (anode) materials for SCs. However, to achieve better performance in SCs, anode materials also play a crucial role [15]. There are some reports on negative electrode materials for SCs, such as that of Zhang et al., which reported a novel anode material with well-dispersed Fe_2_O_3_ decorated on vertically aligned carbon nanotubes (Fe_2_O_3_/VACNT). The Fe_2_O_3_/VACNT was synthesized through a facile two-step hydrothermal method. The Fe_2_O_3_/VACNT composites exhibited a high specific capacitance of 248 F g^−1^ at 8 A g^−1^ in 2M KOH in a negative potential window (−1.2 and 0 V vs. SCE) [16]. Sun et al. synthesized the V_2_O_5_/VACNTs (VN) composite anode, which displayed a high specific capacitance of 284 F g^−1^ in a 1M Na_2_SO_4_ aqueous solution operated in a negative potential window of (−1.1 to 0.0 V) at 2 A g^−1^ [17]. Zhao et al. synthesized the V_2_CT_x_ anode, which revealed a high specific capacitance of 481 F g^−1^ at 1 A g^−1^ and decent cycling stability for up to 60,000 cycles in the 2M ZnSO_4_ aqueous electrolyte [18]. Yang et al. reported the synthesis of ZnFe_2_O_4_/carbon nanofibers via the electrospinning approach, and it showed good specific capacitance 237 F g^−1^ at 1 A g^−1^, with 88.2% capacitance retention. Zhu et al. studied porous ZnFe_2_O_4_ microspheres for SCs, and their results revealed that the ZnFe_2_O_4_ electrode possessed a specific capacitance of 131 F g^−1^, with stable cycle performance up to 92% after 1000 cycles [19]. Despite all these promising results and remarkable advancements in this area, ZnFe_2_O_4_ still has low performance rate, poor Coulombic efficiency, and poor cycling stability because of its large particle size, low intrinsic conductivity, and unstable microstructure [20]. Therefore, to make ZnFe_2_O_4_ with high stability, it is crucial to design high-quality nanostructures with better charge storage capacity.

Herein, we present the synthesis of the ZnFe_2_O_4_ nanoflakes with a binder-free strategy via the hydrothermal method. The ZnFe_2_O_4_ nanoflake’s porous architecture provided a fast transmission of electrolyte ions with abundant active sites. The as-fabricated ZFO-NF@CC electrode exhibited a capacitance of 509 F g^−1^ at 1.5 A g^−1^ with high capacitive storage charge contributions (68%) at 10 mV s^−1^. Additionally, a ZFO-NF@CC||AC@CC asymmetric SC was fabricated using ZFO-NF@CC as an anode and AC@CC as a cathode with a KOH electrolyte. The ZFO-NF@CC||AC@CC attained a high voltage window of 1.8 V and delivered an excellent specific capacitance of 122.2 F g^−1^ at a current density of 2 A g^−1^, a superior energy density of 55.044 Wh kg^−1^ at 1801.44 W kg^−1^, as well as a remarkable retention rate of 96.5% even after 4000 cycles. These findings will open new avenues for developing safe and reliable negative electrodes for high-electrochemical performance SCs.

## 2. Experimental

### 2.1. Materials

Zinc nitrate (Zn(NO_3_)_3_·9H_2_O), iron nitrate (Fe_3_(NO_3_)_3_·9H_2_O), urea, and carbon cloth were acquired from Sigma-Aldrich (St. Louis, MO, USA) and were of analytical grade; they were used without additional purification.

### 2.2. Synthesis of ZnFe_2_O_4_ Nanoflakes

**Figure 1:**
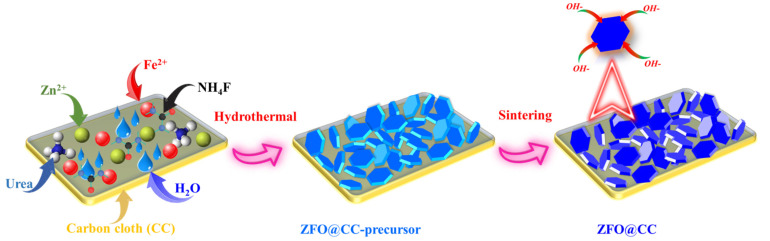
Schematic diagram illustrates the formation mechanism for ZnFe_2_O_4_ nanoflakes at CC.

The ZnFe_2_O_4_ nanoflakes were prepared through the hydrothermal route. Following the typical procedure, 2.5 mmol of Zn(NO_3_)_2_·6H_2_O, 5.0 mmol of Fe_3_(NO_3_)_3_·9H_2_O, and 9 mmol urea were mixed in 80 mL deionized water. Then, the obtained solution was poured into a Teflon-lined autoclave along with a cleansed piece of CC (2 × 2 cm^2^) and heated at 130 °C for 20 h. Afterward, the autoclave was taken out and cooled down at room temperature. The obtained ZFO-NF@CC and the remaining specimens were cleaned using water/ethanol and then dried at 90 °C for 12 h. In the last step, the as-prepared ZFO-NF@CC was sintered at 300 °C for 2 h in the air atmosphere with pure phase ZnFe_2_O_4_ product. The schematic illustration for the fabrication procedure of ZFO-NF@CC is shown in Figure 1.

### 2.3. Fabrication of Single Electrode and Asymmetric Supercapacitor Device

The binder-free ZFO-NF@CC electrode was used directly as a working electrode (1 × 1 cm^2^) with a mass loading density of 1.3 ± 0.1 mg cm^−2^. The activated carbon (AC) supported on the CC substrate was fabricated using the slurry casting method and acted as a positive electrode. The slurry was made by mixing the AC, acetylene black, and PVDF with an 80%:10%:10% ratio and blade cast on the CC. The ZFO-NF@CC||AC@CC SC device (two electrode systems) was simply assembled using the as-fabricated ZFO-NF@CC as the anode and AC@CC as the cathode in a 3 M KOH aqueous electrolyte.

### 2.4. Morphological Characterization of ZnFe_2_O_4_ Nanoflakes

The microstructural and surface morphological investigations of the as-prepared samples were studied using a field-emission scanning electron microscope (FESEM, HITACHI SU8220, Tokyo, Japan) and a transmission electron microscope (TEM JEM-2100F, JEOL, Tokyo, Japan). An X-ray diffractometer (X’Pert Pro PANalytical, Malvern, UK) with Cu-Kα radiations (wavelength λ of about 0.15406 nm) over a 2θ range of 10°–90° was used to investigate the crystal structure of ZnFe_2_O_4_ nanoflakes. The Raman (HJY Lab, RAM Aramics 70, France) and X-ray photoelectron spectroscopy (XPS, Kratos Axis Ultra DLD, Manchester, UK) were used to study the chemical bonds and chemical states of ZnFe_2_O_4_, respectively. The BET and BJH analysis curves were obtained to study specific surface areas and pore sizes with an analyzer (Micromeritics ASAP2460, Norcross, GA, USA).

### 2.5. Electrochemical Characterization

The electrochemical investigation of the sample was examined with cyclic voltammetry (CV), galvanostatic charge–discharge (GCD), and electrochemical impedance spectroscopy (EIS) tests through an electrochemical workstation (CHI 660E, Shanghai, China). The working electrode, counter electrode, and reference electrodes were ZFO-NF@C, a Pt wire, and Ag/AgCl, respectively, in a 3 M KOH aqueous electrolyte. Using different current densities (1.5–20 A/g), a voltage window of −1.0 to 0.0 V was used for CV and GCD tests. The EIS investigations were evaluated over a 0.001–100 kHz frequency range.

## 3. Results and Discussion

**Figure 2:**
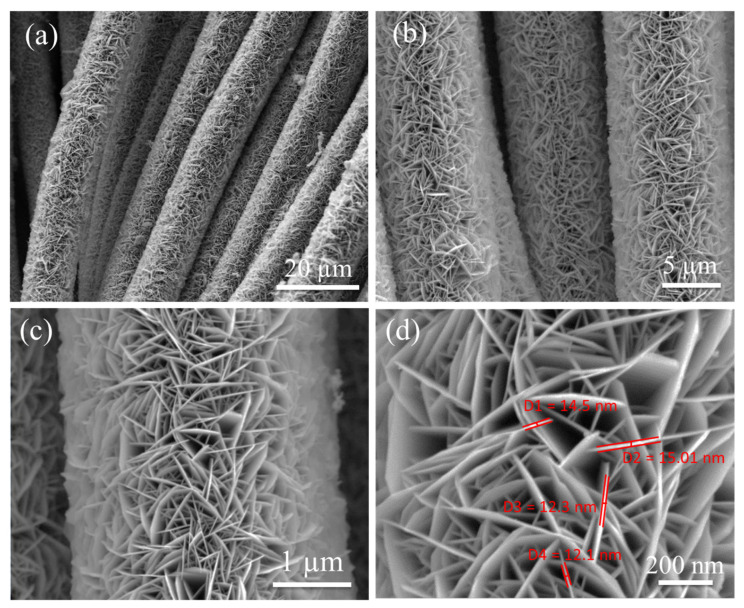
Representative low-resolution FESEM images: (**a**,**b**) vertical nature of ZnFe_2_O_4_ nanoflakes at CC; (**c**) high-resolution image; (**d**) enlarged view.

**Figure 3:**
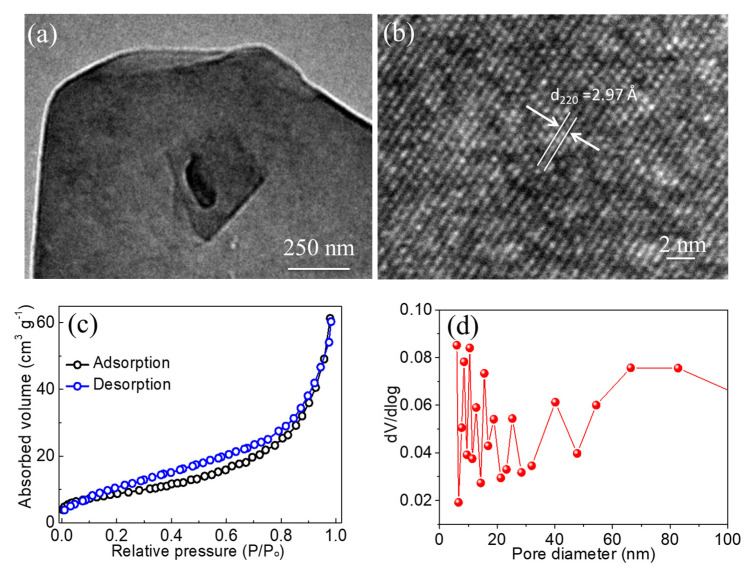
(**a**) TEM image of ZnFe_2_O_4_ at low resolution; (**b**) high-resolution TEM image; (**c**) N_2_ adsorption and desorption isotherms; (**d**) BJH pore size distribution.

The morphological characteristics of ZFO-NF@CC were investigated using FESEM at various resolutions (Figure 2). The low-resolution view (Figure 2a) of ZFO-NF@CC demonstrates the upright texture of nanoflakes densely grown on the fibers of CC. Figure 2b presents the high-resolution FESEM image, which exhibits the small cavities evenly spread and distributed all over the nanoflakes. Furthermore, as shown in the higher-resolution image (Figure 2c), the development of ZFO-NF@CC was confirmed with uniformly small, rich cavities making a highly favorable structure for electrolyte ion accumulation. The highest-resolution FESEM image (Figure 2d) shows that the average thickness of the nanoflakes was around ~13.3 nm. The small assembly of nanoflakes with cavities can be beneficial for the transportation of electrons and reduce the electrode/electrolyte interface resistance. The TEM characterization of ZFO-NF is presented in Figure 3. Figure 3a shows a low-resolution TEM image, which demonstrates the cavity-based structure of ZFO-NF. The high-resolution TEM image of ZFO-NF (Figure 3b) shows that the interplanar spacing was 0.297 nm corresponding to the (220) planes. Well-defined lattice fringes correspond to the high crystalline cubic spinel phase of ZFO-NF. Consequently, ZFO-NF@CC can be favorable to enhance the stability and improve the electrochemical performance as a negative electrode material of the SC.

The Brunauer–Emmett–Teller (BET) measurement technique was employed to examine the porous structure, and an N_2_ adsorption/desorption isotherm was used to present the surface area of ZFO-NF (Figure 3c). The characteristic hysteresis loop from a smaller to a larger value on the relative pressure corresponded to the classical Langmuir IV isotherm. The H_3_ hysteresis loop was located in the middle of the relative pressure, which indicated the existence of mesoporous and macroporous structures in the sample [21]. From the desorption isotherm, the specific surface area of ZFO-NF was assessed to be 46 m^2^ g^−1^. The Barret–Joyner–Halenda (BJH) pore size distribution (Figure 3d) shows a randomly distributed range of pore diameters, demonstrating the existence of mesoporous and macroporous features. These outcomes confirmed the porous characteristic of ZFO-NF. Consequently, they indicated that these porous structures enhanced ion diffusion.

**Figure 4:**
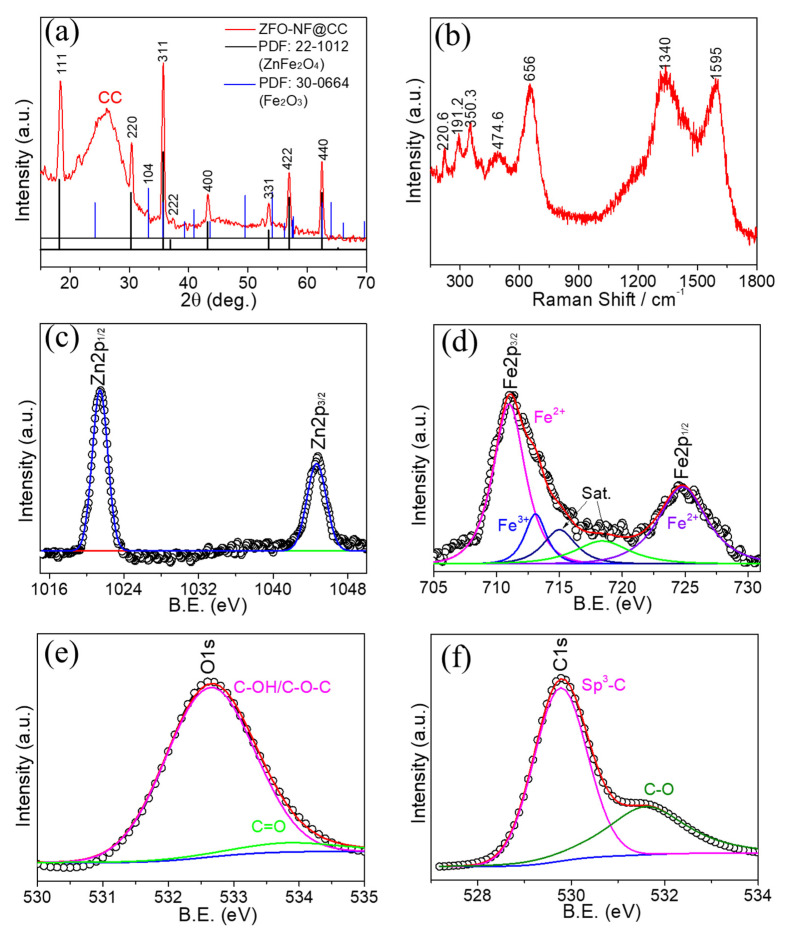
(**a**) XRD pattern; (**b**) Raman spectra; XPS principal spectra of (**c**) Zn 2p, (**d**) Fe 2p, (**e**) O 1s, and (**f**) C 1 s for of ZnFe_2_O_4_ nanoflakes at CC.

The XRD pattern of ZFO-NF@CC is presented in Figure 4a. All the diffraction peaks of ZFO-NF@CC were well-matched with a JCPDS card (22-1012) and appeared at 2θ values of 18.15, 30.3, 35.74, 36.9, 43.2. 53.5, 56.9, and 62.5 corresponding to (311), (220), (311), (222), (400), (331), (422), and (440), respectively, suggesting the cubical spinel phase ZnFe_2_O_4_ [22] of the Fd-3m space group. However, there was one peak (104) that corresponded to the hematite phase of Fe_2_O_3_ (marked with the blue line). The general chemical composition of spinel ferrite is AFe_2_O_4_ with the Fd-3m space group, where A is a divalent cation, which is Zn in our case. The ZnFe_2_O_4_ unit cell contains 32 O atoms in cubic closest-packing, 8 tetrahedral, and 16 octahedral sites, hosting Fe and Zn ions. In the ideal “normal” spinel structure, the Zn ions make fourfold coordination, whereas Fe occupies the octahedral sites [23,24]. Further, the X-ray diffraction peaks were sharp and robust, indicating that the ZnFe_2_O_4_ sample was crystalline.

The structural properties of the as-prepared ZFO-NF@CC sample were studied via Raman spectroscopy. Figure 4b displays that the five peaks for ZFO-NF were located at 220.6, 191.2, 350.3, 474.6, and 656 cm^−1^ and were associated with (A_1g_, E_g_, 3F_2g_) five active Raman modes, which reflected the spinel structure of ZnFe_2_O_4_ with the Fd-3m space group [25], confirming the formation of ZFO-NF, consistent with XRD as well as TEM studies. In addition, CC showed two sharp peaks at 1340 and 1595 cm^−1^ related to the defect peak D and electronic configuration sp^2^ of the graphite carbon peak G, and the ratio of their intensities I_D_/I_G_ was 0.84 for CC [26].

The oxidation states of the ZFO-NF sample were observed using XPS measurements, as depicted in Figure 4. Figure 4c presents the XPS core spectrum of Zn 2p, where two strong peaks were attributed to Zn 2p_3/2_ (at 1044.6 eV), and Zn 2p_1/2_ (1021.5 eV), respectively. The energy difference in the spin–orbit splitting between Zn 2p_3/2_ and Zn 2p_1/2_ was 23.1 eV [27]. These peaks were attributed to the existence of the Zn^2+^ oxidation state. The Fe 2p core spectrum shown in Figure 4d signified the two major binding energy signals assigned to Fe 2p_3/2_ (at 711.1 eV) and Fe 2p_1/2_ (at 724.7 eV). The deconvoluted signals at 718.3 eV indicated the existence of Fe^3+^ and Fe^2+^ oxidation states. In addition to these signals, some weak signals were also seen in the main spectrum of both Zn and Fe, which replicated the presence of satellite signals. The O 1s high-resolution XPS image (Figure 4e) shows a sharp signal at 532.6 eV corresponding to the oxygen bonding, which indicated the existence of the C–OH/C–O–C group, and a weak signal at 533.7 eV was attributed to a C=O group [28]. Figure 4f reveals the high-resolution deconvoluted C 1s spectrum. The binding energy signals at 529.8 eV and 531.6 eV were associated with sp^3^-C as well as C–O bonds, respectively. The XPS results provided evidence of the presence of the Zn^2+^ oxidation state of Zn, as well as the Fe^3+^ and Fe^2+^ oxidation states of Fe, which also confirmed the formation of the ZnFe_2_O_4_ composite. Based on the XPS, the elemental ratio was 14.6: 29.3: 55.1% for Zn 2p, Fe 2p, and O 1s, respectively.

**Figure 5:**
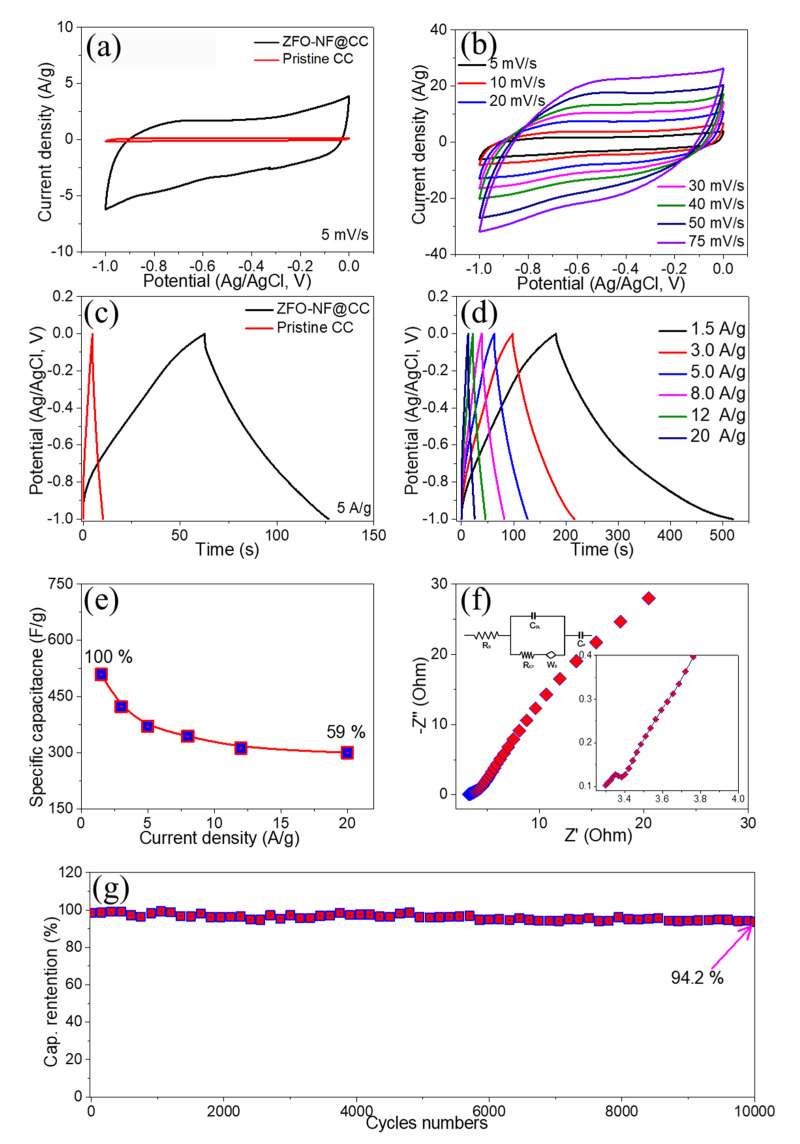
Electrochemical performance of ZnFe_2_O_4_ nanoflakes: (**a**) comparative CV and (**b**) GCD curves of pristine CC and ZFO-NF@CC; (**c**) CV curves; (**d**) GCD curves; (**e**) specific capacitance versus current density; (**f**) Nyquist plot of EIS; (**g**) capacitance retention versus number of cycles.

The electrochemical performance of the ZFO-NF@CC electrode was determined in a 3M KOH electrolyte using a three-electrode system. As a control, the CV curves of pristine CC were executed to investigate the influence of CC on the ZFO-NF@CC electrode under the same conditions and potential window. It can be verified from Figure 5a that the pristine CC had a negligible influence on the performance of the ZFO-NF@CC electrode; thus, such a tiny contribution could be ignored. The CV curves of the ZFO-NF@CC electrode were investigated at various scan rates (5 to 75 mV s^−1^) in a voltage window of 0.0 to −1.0 V, as presented in Figure 5b. With an increase in the scan rate, the CV profiles were shaped almost rectangular at lower and higher scan rates without any deformation, demonstrating that the composite electrode had sufficient storage capacity and rapid charge transport inside and implying a pseudocapacitive charge storage feature [29].

The comparative GCD curves of pristine CC and ZFO-NF@CC electrode using the same voltage window (0.0 to −1.0 V) at a current density of 5 A g^−1^ is shown in Figure 5c. The discharging time of CC was very low compared with that of the ZFO-NF@CC electrode, which showed that the capacitance contribution from pristine CC was very low. These results were in line with CV analysis. The GCD curves of the ZFO-NF@CC electrode were further excited in the 0.0 to −1.0 V at various current densities ranging from 1.5 to 20 A g^−1^, as shown in Figure 5d. The triangular GCD curves with their symmetric characteristic throughout the charging/discharging process demonstrated the pseudocapacitive behavior and were utterly consistent with the CV profiles. The longer discharge time than charge time indicated superior Coulombic efficiency, even greater than 100% at low current densities; such high Coulombic efficiency rates (>100%) have been observed by various research groups in nitrogen-doped graphene and carbon-based materials due to the electrochemical adjustment between electrode and electrolytes [30]. It is further anticipated that, during the first few cycles, electrolyte pores remain plugged, slowing ion discharge, which results in increasing the Coulombic efficiency [31]. At different current densities, the rapid charge transfers and fast I–V response can be attributed to the noticeable decrease in the internal resistance of ZFO-NF@CC. Using the following equation, the specific capacitance was found via galvanostatic discharge curves:

C = IΔt/ΔV_m_ (1)

Here, C denotes the specific capacitance, I denotes the discharge current, ∆t represents the discharge time, m shows the active mass, and ∆V shows the voltage window.

The specific capacitance varied with the current density of ZFO-NF@CC (Figure 5e). The composite material achieved specific capacitance values of 509, 423, 370, 344, 312, and 300 F g^−1^, corresponding to the current density values of 1.5, 3, 5, 8, 12, and 20 A g^−1^, respectively. The ZFO-NF@CC composite exhibited a high-rate capability of 59% from its starting value at 20 A g^−1^. By increasing the current density, the rate capability decreased because of the less scanning time for diffusing the electrolyte ions into the electrode surface. This indicates that at higher current density values, only a small number of ions could reach the inner part of the electrode material. Figure 5f reveals the Nyquist plot of the ZFO-NF@CC electrode, which can be split into two segments: a linear tail in the low-frequency and an arc in the high-frequency range. The intercept at the high-frequency region is designated as equivalent series resistance (R_esr_), such as the electrode/electrolyte interface resistance, the internal resistance of active material, and the electrolytic solution resistance. The semicircle at the high-frequency segment is designated as the Faradaic charge transfer resistance (R_ct_) [32]. The low-frequency region corresponds to the Warburg element designated as electrolytic ion diffusion resistance. The inset of Figure 5d shows that the calculated values of equivalent series resistance (R_esr_ = 3.25 Ω) and charge transfer resistance (R_ct_ = 0.15 Ω) were minimal, indicating better electrical conductivity and good electrochemical kinetics [7]. A long lifetime indicating electrochemical stability is essential for SC’s electrode materials. The cycling performance test of the ZFO-NF@CC electrode at a current density of 20 A/g showed that the ZFO-NF@CC electrode retained 93.6% of the initial capacitance after 10,000 GCD cycles, as displayed in Figure 5g, suggesting its suitability as an energy storage application. Its high capacitance retention was attributed to the suitable electrical contact of the active material with substrate throughout the running time.

**Figure 6:**
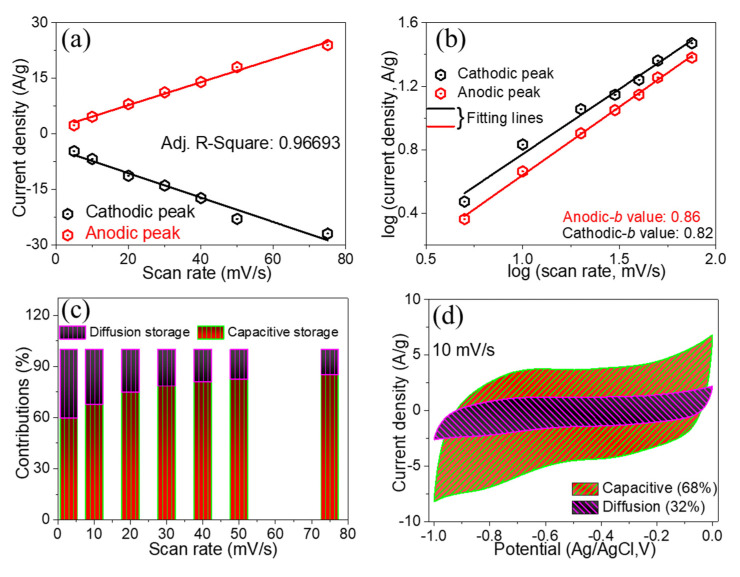
(**a**) Current density as a function of scan rate; (**b**) log (current density) vs. log (scan rate); (**c**) percentage contributions as a function of scan rate; (**d**) CV curve with percentage capacitive and diffusion charge storage at scan rate of 10 mV/s.

The capacitive charge storage mechanism was investigated using the straight-line relationship of the anodic and cathodic current densities with scan rates (Figure 6a). The power law is governed by the following equation to perform the kinetic analysis of the ZFO-NF@CC electrode [33]:

*i*(*V*) = *av^b^* (2)

Here, *i* shows the peak current density, *v* shows the scan rate, and *a* and *b* denote arbitrary constants. The *b*-value can be determined by the slope of log(*v*) vs. log(*i*). The process is diffusion-controlled if the *b*-value is equal to 0.5, and when the *b*-value approaches 1, it corresponds to the surface-controlled process.

log(*i*) = *b*log(*v*) + log(*a*) (3)

Figure 6b displays the plot of log(*i*) as a function of log(*v*). Both the anodic and cathodic values varied linearly, and 0.84 was the anodic *b*-value, while 0.82 was the cathodic *b*-value. Figure 6c illustrates the diffusion-controlled capacity and capacitive capacity contributions of the ZFO-NF@CC electrodes at different scan rates. By varying the scan rates, the charge storage contribution of the capacitive process gradually increased. With the following equation, the diffusion-controlled and capacitive storage processes of ZFO-NF@CC can be estimated:

*i*(*V*) = *k*_1_*v* + *k*_2_*v*^½^ (4)

where the term *k*_1_*v* is the capacitive charge storage, *k*_2_*v*^½^ is the diffusion-controlled charge storage, and *k*_1_ and *k*_2_ denote arbitrary constants. Figure 6d displays that at a 10 mV/s scan rate, the capacitive charge storage achieved 68%, whereas the diffusion-controlled storage attained 32% of the total charge stored. Hence, the capacitive charge storage was the dominant process over the diffusion-controlled storage, with a large specific surface region. Thus, the observed superior capacitive contributions signified the high-rate capability and fast energy storage kinetics of the ZFO-NF@CC electrode [34]. Table 1 shows a comparison of the electrochemical performance indicators of the ZFO-NF@CC electrode with previously reported results.

**Figure 7:**
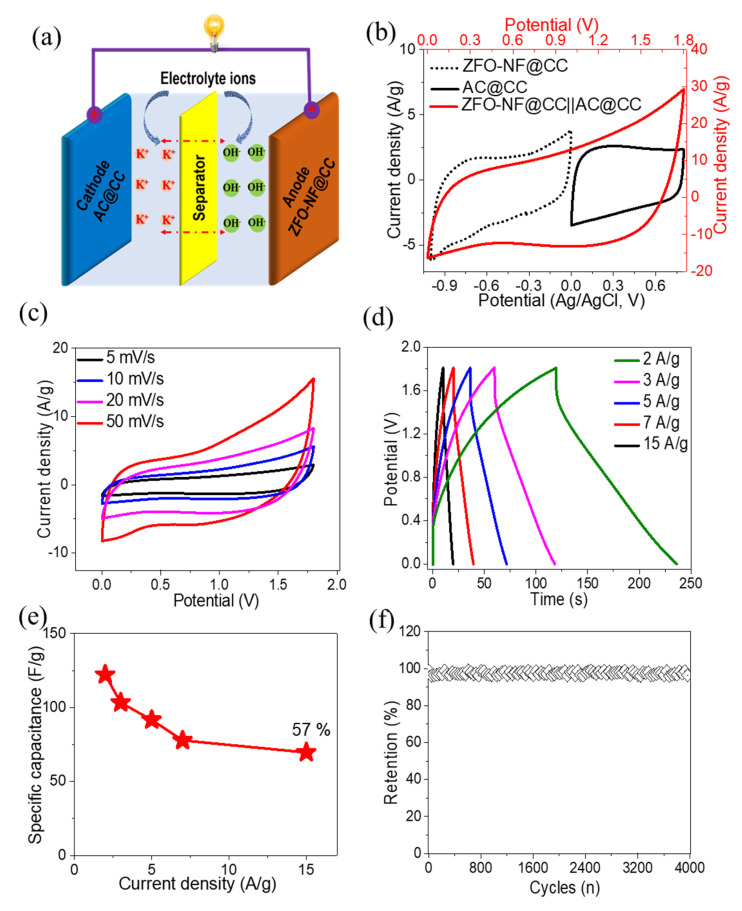
(**a**) Schematic representation of ZFO-NF@CC||AC@CC-HSC device; (**b**) CV curves of ZFO-NF@CC, AC@CC electrodes, and ZFO-NF@CC||AC@CC-HSC device; (**c**) CV curves of ZFO-NF@CC||AC@CC-HSC device at different scan rates from 5 to 50 mV/s; (**d**) GCD curves of ZFO-NF@CC||AC@CC-HSC device at different current densities from 2 to 15 A/g; (**e**) specific capacitance of ZFO-NF@CC||AC@CC-HSC device at various current densities; (**f**) cycling performance of ZFO-NF@CC||AC@CC-HSC device.

In order to study the practical applications of ZFO-NF@CC as an anode, a ZFO-NF@CC||AC@CC device was fabricated using AC@CC as a cathode and a KOH-based aqueous electrolyte. The fabrication process and charge storage mechanism of a ZFO-NF@CC||AC@CC is illustrated in the schematic Figure 7a. The ZFO-NF@CC||AC@CC is expected to be a promising energy storage device, inspired by the performance of the anode and cathode in the aqueous electrolyte. The CV curves of the anode, cathode, and ZFO-NF@CC||AC@CC SC in corresponding voltage windows are exhibited in Figure 7b. The as-fabricated ZFO-NF@CC||AC@CC SC was able to reach the larger voltage window of 0–1.8 V, suggesting energy storage characteristics. The CV curves of ZFO-NF@CC||AC@CC at various scan rates (5 to 50 mV s^−1^) in the potential window of 0–1.8 V are illustrated (Figure 7c). The same shapes of the CV curves at both high as well as low scan rates provide evidence of the device’s excellent pseudocapacitive response and reversible rapid charge storage capability. Figure 7d exhibits the GCD curves of the ZFO-NF@CC||AC@CC device in the same voltage window range as CVs and at various current densities. The almost symmetric GCD curves proved the device’s excellent pseudocapacitive response. The specific capacitance of the ZFO-NF@CC||AC@CC device was calculated by the area under the discharge curve. The specific capacity varied with current density, which is illustrated in Figure 7e. The specific capacitance values were as high as 122.2, 103.3, 91.7, 77.8, and 69.2 F g^−1^ at 2, 3, 5, 7, 15 A g^−1^, respectively, and the specific capacitance retained 57% of its initial value when the current density increased from 2 to 15 A g^−1^, indicating excellent rate capability. The cyclic stability of ZFO-NF@CC||AC@CC attained about 96.5% of the retention rate even after 4000 GCD cycles (Figure 7f). This demonstrates its excellent performance in energy storage applications. In addition, in order to investigate the practical strength of the ZFO-NF@CC||AC@CC device, the energy density E (Wh kg^−1^) and power density P (W kg^−1^) were found using Equations (5) and (6), respectively.

(5) $$E=0.139{\;\mathrm{CV}}^{2}$$

(6) $$P=\frac{E}{\Delta t}\times \left(\frac{1000}{3600}\right)$$

**Figure 8:**
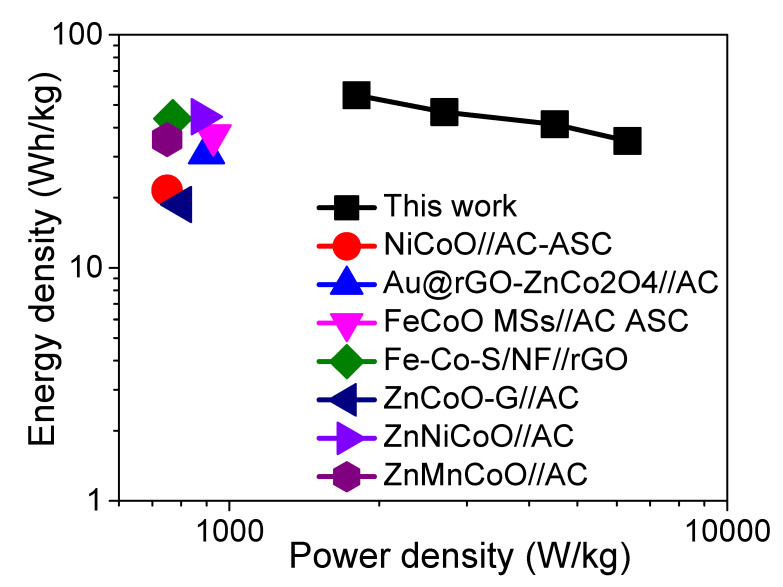
Ragone plot of ZFO-NF@CC||AC@CC-HSC and comparison with recently reported work.

The results are illustrated in the Ragone plot (Figure 8). The energy density of the ZFO-NF@CC||AC@CC device reached its maximum value of 55.044 Wh/kg at 1801.44 W kg^−1^. This is ultrahigh as compared to other energy and power densities reported in the results of ASCs based on bimetallic oxides such as NiCo_2_O_4_//AC-ASC (21.5 Wh kg^−1^ at 750 W kg^−1^) [40]; Au@rGO-ZnCo_2_O_4_//AC (31 Wh kg^−1^ at 900 W kg^−1^) [10]; FeCo_2_O_4_MSs//AC-ASC (37 Wh kg^−1^ at 928 W kg^−1^) [41]; Fe-Co-S/NF//rGO (43.6 Wh kg^−1^ at 770 W kg^−1^) [42]; ZnCoO-G//AC (18.7 Wh kg^−1^ at 800 W kg^−1^) [43]; Zn-Ni-Co-oxide (flowerlike)//AC (44.5 Wh kg^−1^ at 880 W kg^−1^) [44]; and Zn-Mn-Co oxide//AC (35.5 Wh kg^−1^ at 750 W kg^−1^) [45].

## 4. Conclusions

In summary, the binder-free ZFO-NF@CC composite was successfully prepared using a rapid and straightforward hydrothermal method, and its properties were studied via analytical techniques such as XRD, SEM, TEM, Raman, BET, and XPS. The electrochemical performance of the ZFO-NF@CC electrode was conducted as a negative electrode material using CV and GCD for supercapacitor applications. The results revealed the optimum electrochemical performance of the device, with a specific capacitance of 509 F g^−1^ at 1.5 A g^−1^ and 94.2% capacitance retention over 10,000 cycles. Furthermore, the ZFO-NF@CC electrode showed an outstanding charge storage rate of 68% at a scan rate of 10 mV s^−1^. The as-fabricated ZFO-NF@CC||AC@CC showed a relatively high specific capacitance (122.2 F g^−1^ at 2 A g^−1^), with an excellent energy density of 55.044 Wh kg^−1^ at 1801.44 W kg^−1^ and a remarkable capacitance retention rate of 96.5% over 4000 GCD cycles. Overall, such outstanding rate performance was attained in both single electrodes and in the SC device, which can be associated with the rich cavities of ZFO-NF@CC, making a highly favorable structure for electrolyte ion accumulation and rich reaction sites. The obtained results demonstrate that the synthesized ZFO-NF@CC can be the best choice and a promising material to be used in SCs as negative electrodes with high-performance electrochemical properties. 

## Figures and Tables

**Table 1:** Comparative study of specific capacitance values already reported for ZnFe_2_O_4_-based electrodes with those found in the present work.

Sr. No.	Electrode Materials	Electrolyte	Specific Capacitance (F g^−1^)	Current Density (A g^−1^)	No. of Cycles (*n*)	Retention Rate (%)	References
1	ZnFe_2_O_4_ nanoflakes	KOH	509	1.5	10,000	93.5	This work
2	ZnFe_2_O_4_ thin film	NaOH	471	1	1000	72	[35]
3	ZnFe_2_O_4_/carbon	KOH	237	1	1000	88.2	[36]
4	ZnFe_2_O_4_/CNTs	KOH	282	1	5000	56.73	[34]
5	ZnFe_2_O_4_ powder/replica	KOH	279.4	1	1000	80	[20]
6	ZnFe_2_O_4_ nanoparticles	KOH	244	0.5	5000	83.8	[37]
7	ZnFe_2_O_4_ microspheres	KOH	131	1	1000	92	[19]
8	ZnFe_2_O_4_/rGO	KOH	352.9	1	10,000	92.3	[38]
9	ZnFe_2_O_4_/Nanofiber	KOH	408	1	3000	93	[25]
10	ZnFe_2_O_4_ nanoparticles	KOH	474	1	20,000	90	[39]

## Data Availability

Not applicable.

## References

[1] Kyeremateng N.A., Brousse T., Pech D. (2017). Microsupercapacitors as miniaturized energy-storage components for on-chip electronics. Nat. Nanotechnol..

[2] Javed M.S., Shah S.S.A., Najam T., Siyal S.H., Hussain S., Saleem M., Zhao Z., Mai W.J.N.E. (2020). Achieving high-energy density and superior cyclic stability in flexible and lightweight pseudocapacitor through synergic effects of binder-free CoGa_2_O_4_ 2D-hexagonal nanoplates. Nano Energy.

[3] Ji X., Long X. (2016). A review of the ecological and socioeconomic effects of biofuel and energy policy recommendations. Renew. Sustain. Energy Rev..

[4] Sharma K., Arora A., Tripathi S.K. (2019). Review of supercapacitors: Materials and devices. J. Energy Storage.

[5] Miller J.R., Simon P. (2008). Electrochemical capacitors for energy management. Sci. Mag..

[6] Javed M.S., Zhang X., Ali S., Mateen A., Idrees M., Sajjad M., Batool S., Ahmad A., Imran M., Najam T.J.N.E. (2022). Heterostructured bimetallic–sulfide@ layered Ti_3_C_2_T*_x_*–MXene as a synergistic electrode to realize high-energy-density aqueous hybrid-supercapacitors. Nano Energy.

[7] Javed M.S., Lei H., Li J., Wang Z., Mai W. (2019). Construction of highly dispersed mesoporous bimetallic-sulfide nanoparticles locked in N-doped graphitic carbon nanosheets for high energy density hybrid flexible pseudocapacitors. J. Mater. Chem. A.

[8] Venkatesh K., Muthukutty B., Chen S.-M., Karuppiah C., Amanulla B., Yang C.-C., Ramaraj S.K. (2021). Nanomolar level detection of non-steroidal antiandrogen drug flutamide based on ZnMn_2_O_4_ nanoparticles decorated porous reduced graphene oxide nanocomposite electrode. J. Hazard. Mater..

[9] Sen P., De A. (2010). Electrochemical performances of poly (3,4-ethylenedioxythiophene)–NiFe_2_O_4_ nanocomposite as electrode for supercapacitor. Electrochim. Acta.

[10] Patil S.J., Dubal D.P., Lee D.-W. (2020). Gold nanoparticles decorated rGO-ZnCo_2_O_4_ nanocomposite: A promising positive electrode for high performance hybrid supercapacitors. Chem. Eng. J..

[11] Shi Z., Sun G., Yuan R., Chen W., Wang Z., Zhang L., Zhan K., Zhu M., Yang J., Zhao B. (2022). Scalable fabrication of NiCo_2_O_4_/reduced graphene oxide composites by ultrasonic spray as binder-free electrodes for supercapacitors with ultralong lifetime. J. Mater. Sci. Technol..

[12] Javed M.S., Jiang Z., Yang Q., Wang X., Han X., Zhang C., Gu X., Hu C. (2019). Exploring Li-ion hopping behavior in zinc ferrite and promoting performance for flexible solid-state supercapacitor. Electrochim. Acta.

[13] Devi R., Patra J., Tapadia K., Chang J.-K., Maharana T. (2022). Arrangement of ZnFe_2_O_4_@PPy nanoparticles on carbon cloth for highly efficient symmetric supercapacitor. J. Taiwan Inst. Chem. Eng..

[14] Tiwari N., Kadam S., Ingole R., Kulkarni S. (2022). Facile hydrothermal synthesis of ZnFe_2_O_4_ nanostructures for high-performance supercapacitor application. Ceram. Int..

[15] Zhou X., Cao A.-M., Wan L.-J., Guo Y.-G. (2012). Spin-coated silicon nanoparticle/graphene electrode as a binder-free anode for high-performance lithium-ion batteries. Nano Res..

[16] Zhang W., Zhao B., Yin Y., Yin T., Cheng J., Zhan K., Yan Y., Yang J., Li J. (2016). Fe_2_O_3_-decorated millimeter-long vertically aligned carbon nanotube arrays as advanced anode materials for asymmetric supercapacitors with high energy and power densities. J. Mater. Chem. A.

[17] Sun G., Ren H., Shi Z., Zhang L., Wang Z., Zhan K., Yan Y., Yang J., Zhao B. (2021). V_2_O_5_/vertically-aligned carbon nanotubes as negative electrode for asymmetric supercapacitor in neutral aqueous electrolyte. J. Colloid Interface Sci..

[18] Chen W., Zhang L., Ren H., Miao T., Wang Z., Zhan K., Yang J., Zhao B. (2022). V_2_CT_x_ MXene as novel anode for aqueous asymmetric supercapacitor with superb durability in ZnSO_4_ electrolyte. J. Colloid Interface Sci..

[19] Zhu M., Zhang X., Zhou Y., Zhuo C., Huang J., Li S. (2015). Facile solvothermal synthesis of porous ZnFe_2_O_4_ microspheres for capacitive pseudocapacitors. RSC Adv..

[20] Liu H., Guo Y., Zhang Y., Wu F., Liu Y., Zhang D. (2013). Synthesis and properties of ZnFe_2_O_4_ replica with biological hierarchical structure. Mater. Sci. Eng. B.

[21] Li H.-H., Wu X.-L., Zhang L.-L., Fan C.-Y., Wang H.-F., Li X.-Y., Sun H.-Z., Zhang J.-P., Yan Q. (2016). Carbon-Free Porous Zn_2_GeO_4_ Nanofibers as Advanced Anode Materials for High-Performance Lithium Ion Batteries. ACS Appl. Mater. Interfaces.

[22] Yang S., Han Z., Zheng F., Sun J., Qiao Z., Yang X., Li L., Li C., Song X., Cao B. (2018). ZnFe_2_O_4_ nanoparticles-cotton derived hierarchical porous active carbon fibers for high rate-capability supercapacitor electrodes. Carbon.

[23] Javed M.S., Chen J., Chen L., Xi Y., Zhang C., Wan B., Hu C. (2016). Flexible full-solid state supercapacitors based on zinc sulfide spheres growing on carbon textile with superior charge storage. J. Mater. Chem. A.

[24] Pavese A., Levy D., Hoser A.J.A.M. (2000). Cation distribution in synthetic zinc ferrite (Zn_0.97_Fe_2.02_O_4_) from in situ high-temperature neutron powder diffraction. J. Am. Mineral..

[25] Agyemang F.O., Kim H. (2016). Electrospun ZnFe2O4-based nanofiber composites with enhanced supercapacitive properties. Mater. Sci. Eng. B.

[26] Liu Y., Liu X., Dong W., Zhang L., Kong Q., Wang W. (2017). Efficient adsorption of sulfamethazine onto modified activated carbon: A plausible adsorption mechanism. Sci. Rep..

[27] Wang M., Sun L., Cai J., Huang P., Su Y., Lin C. (2013). A facile hydrothermal deposition of ZnFe_2_O_4_ nanoparticles on TiO_2_ nanotube arrays for enhanced visible light photocatalytic activity. J. Mater. Chem. A.

[28] Chen M., Shen X., Chen K., Wu Q., Zhang P., Zhang X., Diao G. (2016). Nitrogen-doped mesoporous carbon-encapsulation urchin-like Fe_3_O_4_ as anode materials for high performance Li-ions batteries. Electrochim. Acta.

[29] Zheng C., Zhou X., Cao H., Wang G., Liu Z. (2014). Synthesis of porous graphene/activated carbon composite with high packing density and large specific surface area for supercapacitor electrode material. J. Power Sources.

[30] Sarkar S., Akshaya R., Ghosh S. (2020). Nitrogen doped graphene/CuCr_2_O_4_ nanocomposites for supercapacitors application: Effect of nitrogen doping on coulombic efficiency. Electrochim. Acta.

[31] Zheng Q., Kvit A., Cai Z., Ma Z., Gong S. (2017). A freestanding cellulose nanofibril–reduced graphene oxide–molybdenum oxynitride aerogel film electrode for all-solid-state supercapacitors with ultrahigh energy density. J. Mater. Chem. A.

[32] Javed M.S., Jiang Z., Zhang C., Chen L., Hu C., Gu X. (2016). A high-performance flexible solid-state supercapacitor based on Li-ion intercalation into tunnel-structure iron sulfide. Electrochim. Acta.

[33] Javed M.S., Han X., Hu C., Zhou M., Huang Z., Tang X., Gu X. (2016). Tracking Pseudocapacitive Contribution to Superior Energy Storage of MnS Nanoparticles Grown on Carbon Textile. ACS Appl. Mater. Interfaces.

[34] Seong J.-G., Ko T.H., Lei D., Choi W.-K., Kuk Y.-S., Seo M.-K., Kim B.-S. (2021). Engineered NiCo-LDH nanosheets-and ZnFe_2_O_4_ nanocubes-decorated carbon nanofiber bonded mats for high-rate asymmetric supercapacitors. Green Energy Environ..

[35] Raut S.S., Sankapal B.R. (2016). First report on synthesis of ZnFe_2_O_4_ thin film using successive ionic layer adsorption and reaction: Approach towards solid-state symmetric supercapacitor device. Electrochim. Acta.

[36] Yang S., Ai J., Han Z., Zhang L., Zhao D., Wang J., Yang C., Cao B. (2020). Electrospun ZnFe2O4/carbon nanofibers as high-rate supercapacitor electrodes. J. Power Sources.

[37] Li L., Bi H., Gai S., He F., Gao P., Dai Y., Zhang X., Yang D., Zhang M., Yang P. (2017). Uniformly dispersed ZnFe_2_O_4_ nanoparticles on nitrogen-modified graphene for high-performance supercapacitor as electrode. Sci. Rep..

[38] Yang S., Han Z., Sun J., Yang X., Hu X., Li C., Cao B. (2018). Controllable ZnFe_2_O_4_/reduced graphene oxide hybrid for high-performance supercapacitor electrode. Electrochim. Acta.

[39] Yang S., Han Z., Sun J., Yang X., Li C., Wang R., Cao B. (2018). Preparation of defective ZnFe_2_O_4_/graphene composites and their charge storage properties. Electrochem. Commun..

[40] Xu K., Yang J., Hu J. (2018). Synthesis of hollow NiCo_2_O_4_ nanospheres with large specific surface area for asymmetric supercapacitors. J. Colloid Interface Sci..

[41] Li S., Wang Y., Sun J., Xu C., Chen H. (2020). Simple Preparation of Porous FeCo_2_O_4_ Microspheres and Nanosheets for Advanced Asymmetric Supercapacitors. ACS Appl. Energy Mater..

[42] Le K., Gao M., Liu W., Liu J., Wang Z., Wang F., Murugadoss V., Wu S., Ding T., Guo Z. (2019). MOF-derived hierarchical core-shell hollow iron-cobalt sulfides nanoarrays on Ni foam with enhanced electrochemical properties for high energy density asymmetric supercapacitors. Electrochim. Acta.

[43] Yu J., Cui Z., Li X., Chen D., Ji J., Zhang Q., Sui J., Yu L., Dong L. (2019). Facile fabrication of ZIF-derived graphene-based 2D Zn/Co oxide hybrid for high-performance supercapacitors. J. Energy Storage.

[44] Hu W., Wei H., She Y., Tang X., Zhou M., Zang Z., Du J., Gao C., Guo Y., Bao D. (2017). Flower-like nickel-zinc-cobalt mixed metal oxide nanowire arrays for electrochemical capacitor applications. J. Alloys Compd..

[45] Hussain I., Mohamed S.G., Ali A., Abbas N., Ammar S.M., Al Zoubi W. (2019). Uniform growth of Zn-Mn-Co ternary oxide nanoneedles for high-performance energy-storage applications. J. Electroanal. Chem..

